# Estimation of Jacquard’s genetic identity coefficients with bi-allelic variants by constrained least-squares

**DOI:** 10.1038/s41437-024-00731-z

**Published:** 2024-11-07

**Authors:** Jan Graffelman, Bruce S. Weir, Jérôme Goudet

**Affiliations:** 1 Department of Statistics and Operations Research, Universitat Politècnica de Catalunya, Barcelona, Spain; 2 Department of Biostatistics, University of Washington, Seattle, WA USA; 3 Department of Ecology and Evolution, University of Lausanne, Lausanne, Switzerland

**Keywords:** Genetic variation, Consanguinity, Genetic variation, Genetic variation

## Abstract

The Jacquard genetic identity coefficients are of fundamental importance in relatedness research. We address the estimation of these coefficients as well as other relationship parameters that derive from them such as kinship and inbreeding coefficients using a concise matrix framework. Estimation of the Jacquard coefficients via likelihood methods and the expectation–maximization algorithm is computationally very demanding for large numbers of polymorphisms. We propose a constrained least squares approach to estimate the Jacquard coefficients. A simulation study shows constrained least squares achieves root-mean-squared errors that are comparable with those of the maximum likelihood approach, in particular when founder allele frequencies are unknown, while obtaining enormous computational savings.

## Introduction

**Fig. 1:**
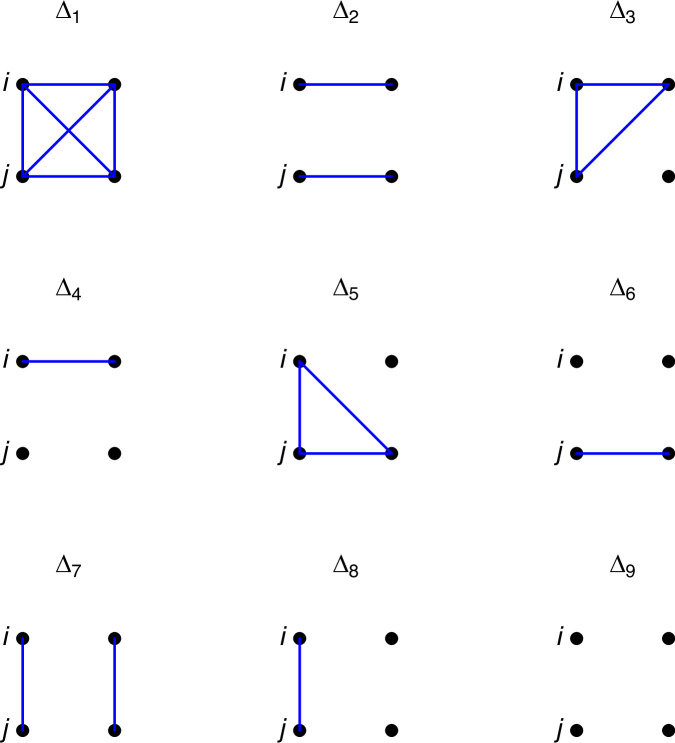
The IBD patterns for a pair (*i*, *j*) of individuals. Dots represent alleles. Blue lines connect pairs of alleles that are IBD.

The estimation of the degree of genetic relatedness, either by using pedigrees or molecular marker data, is of keen interest for a variety of purposes (Weir et al. 2006). It is, among others, useful for establishing genealogies, for paternity testing, and for maintaining genetic diversity in breeding programs with endangered species. Accounting for relatedness is crucial in genetic association studies (Astle and Balding 2009). The definition of the concept of alleles that derive from the same allele in some reference population as identical-by-descent (IBD) alleles (Malécot 1969) was foundational for relatedness research. Harris (1964) enumerated 15 modes of identity-by-descent, which reduce to nine modes if the paternal and maternal origins of the alleles are not distinguished. These modes were represented pictorially by Jacquard (1972; 1974), and are nowadays commonly referred to as Jacquard’s coefficients. Jacquard’s coefficients underlie coancestry coefficients, inbreeding coefficients and other relationship parameters (see section “Theory”) and are practically important in quantitative genetics for estimating non-additive components of variance in inbred populations. Figure 1 shows the nine condensed states where blue lines indicate an IBD relationship between two alleles. Non-horizontal lines show IBD relationships between individuals of a pair, horizontal lines refer to IBD relationships within an individual, i.e., these refer to an inbred state. We use the symbol *Δ*_*k*_ to either refer to the particular mode or its probability. When convenient, we will use ${\Delta }_{k}^{(i,j)}$ to emphasize its pairwise probabilistic nature. For pattern *Δ*_7_ the two individuals share two IBD alleles among them; for pattern *Δ*_1_, the two individuals share one IBD allele across all their four chromosomes; for patterns *Δ*_3_, *Δ*_5_ and *Δ*_8_ they share one, and for the remaining states they share none. States *Δ*_1_ through *Δ*_6_ all refer to inbred states. When there is no inbreeding, the number of states reduces to three (*Δ*_7_, *Δ*_8_ and *Δ*_9_), and their relative probabilities are known as the Cotterman coefficients (1940). Under non-inbred conditions, Thompson (1976) showed that the Cotterman coefficients are limited to a subspace of the two-dimensional three-part simplex, satisfying ${\Delta }_{8}^{2}\ge 4{\Delta }_{7}{\Delta }_{9}$.

Probabilities of observed genotypes for pairs of individuals are readily related to the Jacquard coefficients for given allele probabilities (Cockerham 1971). We will use a 0 and a 1 respectively to represent the major and minor allele at a bi-allelic locus, and use 0/0, 0/1 and 1/1 to represent the corresponding diploid genotypes. Thus, for a bi-allelic variant with minor allele probability (MAP) *p* and major allele probability *q*, the probability of observing either two minor homozygotes or two major homozygotes is, given state *Δ*_1_, *p* or *q* respectively. Likewise, state *Δ*_2_ is compatible only with genotype pairs (0/0,1/1), (0/0,0/0), (1/1,1/1) and (1/1,0/0), which will have probabilities *pq*, *p*^2^, *q*^2^ and *qp* respectively, since the first allele of an individual is necessarily the same as the second. By the same token, for each given mode the joint genotype probabilities can be developed for all nine possible genotype pairs and are given in Table 1.

**Table 1:** The bi-allelic condensed system, consisting of joint genotype probabilities for given IBD patterns and allele probabilities.

No.	Genotype pair	*Δ*_1_	*Δ*_2_	*Δ*_3_	*Δ*_4_	*Δ*_5_	*Δ*_6_	*Δ*_7_	*Δ*_8_	*Δ*_9_
1	(0/0,0/0)	*q*	*q*^2^	*q*^2^	*q*^3^	*q*^2^	*q*^3^	*q*^2^	*q*^3^	*q*^4^
2	(0/0,0/1)	0	0	*pq*	2*pq*^2^	0	0	0	*pq*^2^	2*pq*^3^
3	(0/0,1/1)	0	*pq*	0	*p*^2^*q*	0	*pq*^2^	0	0	*p*^2^*q*^2^
4	(0/1,0/0)	0	0	0	0	*pq*	2*pq*^2^	0	*pq*^2^	2*pq*^3^
5	(0/1,0/1)	0	0	0	0	0	0	2*pq*	*p*^2^*q* + *pq*^2^	4*p*^2^*q*^2^
6	(0/1,1/1)	0	0	0	0	*pq*	2*p*^2^*q*	0	*p*^2^*q*	2*p*^3^*q*
7	(1/1,0/0)	0	*pq*	0	*pq*^2^	0	*p*^2^*q*	0	0	*p*^2^*q*^2^
8	(1/1,0/1)	0	0	*pq*	2*p*^2^*q*	0	0	0	*p*^2^*q*	2*p*^3^*q*
9	(1/1,1/1)	*p*	*p*^2^	*p*^2^	*p*^3^	*p*^2^	*p*^3^	*p*^2^	*p*^3^	*p*^4^

Table 1 has been published in different forms by several authors (Anderson and Weir 2007; Cockerham 1971; Csűrös 2014; Laporte et al. 2017; Wang 2022; Weir et al. 2006), depending on whether or not two or more alleles are considered, and depending on whether the order of the individuals in a pair is taken into account or not. Anderson & Weir (2007) developed a (multi-allelic) extended parametrization of Table 1 in order to allow for population substructure, i.e., allowing for a subpopulation whose allele frequency has drifted away from that in the original parent population. As given here, Table 1 is strictly for the bi-allelic case, the order of the alleles of an individual is considered irrelevant (i.e., heterozygotes 0/1 and 1/0 are not distinguished), but the order of the individuals in a pair is taken into account. A homogeneous population with no differentiation of allele probabilities is assumed throughout.

If we define **g** as the 9 × 1 vector of (marginal) joint genotype probabilities, then by the law of total probability, we have

1 $${\bf{g}}=\mathop{\sum }\limits_{i=1}^{9}P\left({\bf{g}}| {\Delta }_{i}\right){\Delta }_{i}={\bf{M}}{\mathbf{\Delta }}.$$

where **M** is the 9 × 9 matrix given in Table 1, whose entries are determined by a single parameter, the minor allele probability, and ${\mathbf{\Delta }}={({\Delta }_{1},{\Delta }_{2},\ldots ,{\Delta }_{9})}^{{\prime} }$ the column vector containing the coefficients for one particular pair (*i*, *j*) of individuals. Alternative parametrizations of the system in terms of genetic correlations coefficients (Ackerman et al. 2017) are possible, but not considered here. Equation (1) refers to the so-called condensed coefficients (Jacquard 1974), and we will refer to it as the *bi-allelic condensed system*.

Up to only a few years ago, much of relatedness research mostly focused on the estimation of the Cotterman coefficients and the derived kinship coefficient, where the latter is defined as the probability that two alleles, each taken at random from an individual, are IBD. The estimation of the IBD coefficients by maximum likelihood (Thompson 1975), assuming non-inbred individuals and known allele probabilities, was a milestone achievement for relatedness research. Under absence of inbreeding, the kinship coefficient is obtained from the Cotterman coefficients as ${\theta }_{1}=\frac{1}{2}{\Delta }_{7}+\frac{1}{4}{\Delta }_{8}$. Milligan (2003) performed an extensive study comparing the different estimators of the kinship coefficient, and generally recommended the maximum likelihood estimator across a broad spectrum of conditions. Over the last decade, interest for the estimation of the full set of Jacquard coefficients has increased (Guan and Levy 2024; Hanghøj et al. 2019; Korneliussen and Moltke 2015; Zheng et al. 2012). Nowadays, the ever-growing amount of available genetic information and computational resources have lead to an increased interest in the estimation of relationship parameters such as coancestry, individual inbreeding coefficients, and others. Multiple estimators have recently been proposed for these parameters, including allele-sharing estimators (Goudet et al. 2018; Weir and Goudet 2017) for coancestry and inbreeding that account for genetic sampling (Weir 1996). Most relationship parameters of interest can be derived from Jacquard’s coefficients. For family-based studies, efficient algorithms are available that allow for the calculation of the theoretical Jacquard coefficients according to a specified pedigree (Abney 2009; Karigl 1981; Lange and Sinsheimer 1992). For population-based genetic studies, pedigrees are often not available, and for those studies with available pedigrees, the latter are known mostly not to be error-free and often incomplete. It is therefore of great interest to estimate the Jacquard coefficients from the molecular marker data. Correspondingly, some attempts have been made to estimate the full set of nine condensed Jacquard coefficients with SNP data, using different methods (Hanghøj et al. 2019; Laporte et al. 2017; Wang 2022), despite the fact that for bi-allelic data, Jacquard’s coefficients have been shown not to be identifiable (Csűrös 2014).

In this article, we propose to estimate the nine Jacquard coefficients and derived quantities by using a constrained least squares (CLS) criterion, as described in section “Theory” below. We perform pedigree-based simulations to compare CLS and EM estimates, and also address the computational cost of scaling up the algorithms to a full genome.

## Theory

### Notation

We first develop our notation. Since many of the quantities involved such as Jacquard coefficients, kinship coefficients and others are pairwise quantities, we have found it convenient to use matrix notation, using bold lowercase and uppercase letters to indicate vectors and matrices respectively. We use a 1 to denote the minor allele of a SNP, and 0 to denote its major allele. We use *p*_*i*_ to refer to minor allele probability of the *i*th SNP and *q*_*i*_ = 1 − *p*_*i*_ for its major allele probability. We use, following Jacquard (1974), the scalar *Δ*_*k*_ to denote the probability of state *k* for a pair of individuals. We will use the scalar ${\Delta }_{k}^{(i,j)}$ to emphasize the coefficient is used for a particular pair (*i*, *j*). For convenience, we store all pairwise coefficients in *n* × *n* subindexed matrices **Δ**_1_, **Δ**_2_, …**Δ**_9_, e.g., the element in row *i* and column *j* of matrix **Δ**_1_ contains the probability *Δ*_1_ for a particular pair of individuals. When convenient, we will make use of vector **Δ** (small case without subscript), and **Δ** = (*Δ*_1_, …, *Δ*_9_) refers to the set of nine Jacquard coefficients for a particular pair. We start by noting that the probabilities of the nine possible states constitute a closed simplex given by

2 $${S}^{9}=\left\{{\Delta }_{1},{\Delta }_{2},\ldots ,{\Delta }_{9}| {\Delta }_{k}\ge 0,\mathop{\sum }\limits_{k=1}^{9}{\Delta }_{k}=1\right\}.$$

Thompson (1978) derived multiple restrictions on the Jacquard coefficients by considering specific subsets of the coefficients, most notably the case of absence of inbreeding (Thompson 1976), which leads to *Δ*_*k*_ = 0 for *k* ≤ 6 and ${\Delta }_{8}^{2}\ge 4{\Delta }_{7}{\Delta }_{9}$. In this article, we will make no use of these restrictions, and allow all Jacquard coefficients to be non-zero. We enumerate some of the well-known quantities that are derived from the Jacquard coefficients, and stress their pairwise nature using superscript (*i*, *j*) to indicate a pair (*i*, *j*); this paves the way for our matrix notation below and clarifies the inbreeding coefficients we use. The coancestry or kinship coefficient is given by

3 $${\theta }_{1}^{(i,j)}={\Delta }_{1}^{(i,j)}+\frac{1}{2}({\Delta }_{3}^{(i,j)}+{\Delta }_{5}^{(i,j)}+{\Delta }_{7}^{(i,j)})+\frac{1}{4}{\Delta }_{8}^{(i,j)}.$$

We define the probability that individual *i* of a given pair (*i*, *j*) carries two copies of the same ancestral allele as:

4 $${\theta }_{2i}^{(i,j)}={\Delta }_{1}^{(i,j)}+{\Delta }_{2}^{(i,j)}+{\Delta }_{3}^{(i,j)}+{\Delta }_{4}^{(i,j)}.$$

Likewise, the probability of this for individual *j* of pair (*i*, *j*) is:

5 $${\theta }_{2j}^{(i,j)}={\Delta }_{1}^{(i,j)}+{\Delta }_{2}^{(i,j)}+{\Delta }_{5}^{(i,j)}+{\Delta }_{6}^{(i,j)}.$$

where we use subscripts 2*i* and 2*j* to refer to individual *i* or *j* of the pair. We stress that the LHSs of Eqs. (4) and (5) sum pairwise quantities and remain pairwise quantities. These quantities have been termed *θ*_2*A*_, *θ*_2*B*_ or *f*_*A*_, *f*_*B*_ by others (Csűrös 2014; Jacquard 1974), and are generally referred to as inbreeding coefficients. We prefer to use the term inbreeding coefficient for a truly individual (non-pairwise) quantity, and consequently obtain these individual inbreeding coefficients as

6 $${\theta }_{2i}^{(i,i)}={\Delta }_{1}^{(i,i)}\quad {\rm{and}}\quad {\theta }_{2j}^{(j,j)}={\Delta }_{1}^{(j,j)},$$

which follows from the nullity of ${\Delta }_{k}^{(i,i)}$ for *k* ∈ (2, 3, 4, 5, 6), and is the scalar equivalent of our matrix equation (18) below. We express (6) more concisely as ${\theta }_{2}^{(i)}={\Delta }_{1}^{(i,i)}$, where the superscript (*i*) indicates this is an individual-level quantity. In brief, we will use ${\theta }_{2}^{(i)}$ to refer to the individual inbreeding coefficient of individual *i* and ${\theta }_{2i}^{(i,j)}$ to refer to the probability that individual *i* of a given pair (*i*, *j*) carries two copies of the same ancestral allele, and use ${\hat{\theta }}_{2}^{(i)}$ and ${\hat{\theta }}_{2i}^{(i,j)}$ to refer to the sample estimators of these quantities.

The probability of at least one pair of IBD alleles among three randomly selected alleles, of the four carried by individuals (*i*, *j*), is given by

7 $${\theta }_{3}^{(i,j)}={\Delta }_{1}^{(i,j)}+{\Delta }_{2}^{(i,j)}+{\Delta }_{3}^{(i,j)}+{\Delta }_{5}^{(i,j)}+{\Delta }_{7}^{(i,j)}+\frac{1}{2}\left({\Delta }_{4}^{(i,j)}+{\Delta }_{6}^{(i,j)}+{\Delta }_{8}^{(i,j)}\right).$$

finally, we define

8 $${\theta }_{4}^{(i,j)}=\frac{1}{2}\left({\Delta }_{4}^{(i,j)}-{\Delta }_{6}^{(i,j)}\right),$$

making for five identifiable relatedness parameters (Csűrös 2014). A statistical model is identifiable if there is a one-to-one correspondence between the values of the parameters of the model and the probability distribution of the data. For bi-allelic polymorphisms, the set of condensed Jacquard coefficients is not identifiable because two different sets of coefficients can generate the same probability distribution of joint genotypes, as illustrated in Appendix A. All five identifiable relatedness parameters above of a pair can be conveniently obtained by a linear transformation (**Q**, of rank five) of the Jacquard coefficients as ***θ*** = **QΔ**, i.e.,

9 $${\boldsymbol{\theta }}=\left[\begin{array}{c}{\theta }_{1}\\ {\theta }_{2i}\\ {\theta }_{2j}\\ {\theta }_{3}\\ {\theta }_{4}\end{array}\right]=\left[\begin{array}{ccccccccc}1&0&\frac{1}{2}&0&\frac{1}{2}&0&\frac{1}{2}&\frac{1}{4}&0\\ 1&1&1&1&0&0&0&0&0\\ 1&1&0&0&1&1&0&0&0\\ 1&1&1&\frac{1}{2}&1&\frac{1}{2}&1&\frac{1}{2}&0\\ 0&0&0&\frac{1}{2}&0&-\frac{1}{2}&0&0&0\end{array}\right]\left[\begin{array}{c}{\Delta }_{1}\\ {\Delta }_{2}\\ {\Delta }_{3}\\ {\Delta }_{4}\\ {\Delta }_{5}\\ {\Delta }_{6}\\ {\Delta }_{7}\\ {\Delta }_{8}\\ {\Delta }_{9}\end{array}\right].$$

We note that the vector of identifiable relatedness parameters ***θ*** is not unique, and that alternative vectors of identifiable relatedness parameters can be obtained by defining linear combinations of the rows of **Q** (Csűrös 2014, Theorems 4 and 5). It is insightful to further develop the matrix notation, and the simplex property implies that

10 $$\mathop{\sum }\limits_{k=1}^{9}{{\mathbf{\Delta }}}_{k}={\bf{J}}={\bf{1}}{{\bf{1}}}^{{\prime} }.$$

Note that for states *Δ*_3_ and *Δ*_5_, an interchange of the two individuals *i* and *j* implies a change from state *Δ*_3_ to *Δ*_5_ for one individual, and a change from state *Δ*_5_ to *Δ*_3_ for the other (see Figure 1). Correspondingly, **Δ**_3_ and **Δ**_5_ are not symmetric but are each other’s mutual transpose. The same holds true for states *Δ*_4_ and *Δ*_6_. For all other states, an interchange of individuals does not bring about a change of state, and we therefore have that

11 $${{\mathbf{\Delta }}}_{3}={{{\mathbf{\Delta }}}_{5}}^{{\prime} },\qquad {{\mathbf{\Delta }}}_{4}={{{\mathbf{\Delta }}}_{6}}^{{\prime} }\quad {\rm{and}}\quad {{\mathbf{\Delta }}}_{k}={{{\mathbf{\Delta }}}_{k}}^{{\prime} }\quad \forall k\in (1,2,7,8,9).$$

When a Jacquard coefficient of an individual with itself is considered, all states have probability zero except *Δ*_1_ and *Δ*_7_, because an individual always shares one or two IBD alleles with itself, either inbred (*Δ*_1_) or not (*Δ*_7_). Consequently, we have

12 $${\rm{diag}}({{\mathbf{\Delta }}}_{1})+{\rm{diag}}({{\mathbf{\Delta }}}_{7})={\bf{1}}\quad {\rm{and}}\quad {\rm{diag}}({{\mathbf{\Delta }}}_{k})={\bf{0}}\quad \forall k\in (2,3,4,5,6,8,9),$$

such that only **Δ**_1_ and **Δ**_7_ can have non-zero diagonals, and where operator diag(⋅) extracts the diagonal of a matrix into a column vector. We next develop matrices for relatedness coefficients. The kinship matrix is defined as

13 $${{\boldsymbol{\theta }}}_{1}={{\mathbf{\Delta }}}_{1}+\frac{1}{2}({{\mathbf{\Delta }}}_{3}+{{\mathbf{\Delta }}}_{5}+{{\mathbf{\Delta }}}_{7})+\frac{1}{4}{{\mathbf{\Delta }}}_{8}.$$

This matrix is symmetric because

14 $${{{\boldsymbol{\theta }}}_{1}}^{{\prime} }={{\mathbf{\Delta }}}_{1}+\frac{1}{2}({{\mathbf{\Delta }}}_{3}^{{\prime} }+{{\mathbf{\Delta }}}_{5}^{{\prime} }+{{\mathbf{\Delta }}}_{7})+\frac{1}{4}{{\mathbf{\Delta }}}_{8}={{\mathbf{\Delta }}}_{1}+\frac{1}{2}({{\mathbf{\Delta }}}_{5}+{{\mathbf{\Delta }}}_{3}+{{\mathbf{\Delta }}}_{7})+\frac{1}{4}{{\mathbf{\Delta }}}_{8}={{\boldsymbol{\theta }}}_{1}.$$

Note that for self-kinship

15 $${\rm{diag}}({{\boldsymbol{\theta }}}_{1})={\rm{diag}}({{\mathbf{\Delta }}}_{1})+\frac{1}{2}{\rm{diag}}({{\mathbf{\Delta }}}_{7}).$$

Matrices of inbreeding coefficients are, according to Equation (4), obtained as

16 $${{\boldsymbol{\theta }}}_{2i}={{\mathbf{\Delta }}}_{1}+{{\mathbf{\Delta }}}_{2}+{{\mathbf{\Delta }}}_{3}+{{\mathbf{\Delta }}}_{4},\quad {\rm{and}}\quad {{\boldsymbol{\theta }}}_{2j}={{\mathbf{\Delta }}}_{1}+{{\mathbf{\Delta }}}_{2}+{{\mathbf{\Delta }}}_{5}+{{\mathbf{\Delta }}}_{6}.$$

So that

17 $${{\boldsymbol{\theta }}}_{2j}={{\mathbf{\Delta }}}_{1}+{{\mathbf{\Delta }}}_{2}+{{\mathbf{\Delta }}}_{3}^{{\prime} }+{{\mathbf{\Delta }}}_{4}^{{\prime} }={{\boldsymbol{\theta }}}_{2i}^{{\prime} },$$

which implies

18 $${\rm{diag}}({{\boldsymbol{\theta }}}_{2j})={\rm{diag}}({{\boldsymbol{\theta }}}_{2i})={\rm{diag}}({{\mathbf{\Delta }}}_{1}).$$

Multiplying (15) by two and combining with (11)

19 $${\rm{diag}}(2{{\boldsymbol{\theta }}}_{1}-{\bf{I}})={\rm{diag}}(2{{\mathbf{\Delta }}}_{1}+{{\mathbf{\Delta }}}_{7}-{\bf{I}})={\rm{diag}}({{\mathbf{\Delta }}}_{1}),$$

which can be rewritten as

20 $${\rm{diag}}({{\boldsymbol{\theta }}}_{1})=\frac{1}{2}{\rm{diag}}({\bf{I}}+{{\mathbf{\Delta }}}_{1}),$$

where the latter equation is the matrix formulation of the well-known result that self-kinship relates to inbreeding (${\theta }_{jj}=\frac{1}{2}(1+{F}_{j})$, in a usual scalar notation). Inbreeding coefficients for each individual are thus obtained as the diagonal elements of **Δ**_1_, or equivalently, as the row means of ***θ***_2*i*_ or the column means of ***θ***_2*j*_. The obvious matrix formulation for *θ*_3_ is

21 $${{\boldsymbol{\theta }}}_{3}={{\mathbf{\Delta }}}_{1}+{{\mathbf{\Delta }}}_{2}+{{\mathbf{\Delta }}}_{3}+{{\mathbf{\Delta }}}_{5}+{{\mathbf{\Delta }}}_{7}+\frac{1}{2}({{\mathbf{\Delta }}}_{4}+{{\mathbf{\Delta }}}_{6}+{{\mathbf{\Delta }}}_{8}),$$

which has diag(***θ***_3_) = **1**, and is symmetric. Finally, for *θ*_4_

22 $${{\boldsymbol{\theta }}}_{4}=\frac{1}{2}({{\mathbf{\Delta }}}_{4}-{{\mathbf{\Delta }}}_{6}),$$

is skew-symmetric (${{\boldsymbol{\theta }}}_{4}^{{\prime} }=-{{\boldsymbol{\theta }}}_{4}$). The aforementioned close relationship between states (*Δ*_3_, *Δ*_5_) and (*Δ*_4_, *Δ*_6_), suggests these states might be joined by summation, reducing the number of parameters to be estimated to seven. This reduction is developed in Appendix B.

Following Thompson (2013), Weir and Goudet (2017) emphasized the relative nature of coancestry and inbreeding, defining the compound quantities of *relative* coancestry and *relative* inbreeding, which we will indicate with *ψ*_1_ and *ψ*_2_ respectively. These quantities are readily obtained from the previous expressions. We define the theoretical average coancestry over all *n*(*n* − 1) pairs as

23 $${\theta }_{S}={{\bf{1}}}^{{\prime} }\left({{\boldsymbol{\theta }}}_{1}\odot {\bf{W}}\right){\bf{1}}/(n(n-1)),$$

where ⊙ represents the Hadamard product (i.e., elementwise multiplication), and **W** a weight matrix of ones with zeros on the diagonal (**W** = **J** − **I**). The symmetric matrix of relative coancestry coefficients **Ψ**_1_, is obtained as

24 $${{\mathbf{\Psi }}}_{1}=\left({{\boldsymbol{\theta }}}_{1}-{\theta }_{S}{\bf{J}}\right)/(1-{\theta }_{S}).$$

We note that **Ψ**_1_ precisely contains the relative individual inbreeding coefficients on its diagonal. Let ***ψ***_2_ be a column vector containing these coefficients. Using Eq. (18) We have that

25 $${{\boldsymbol{\psi }}}_{2}=({\rm{diag}}({{\mathbf{\Delta }}}_{1})-{\bf{1}}{\theta }_{S})/(1-{\theta }_{S})={\rm{diag}}({{\mathbf{\Psi }}}_{1}).$$

In the remainder, we will use ${\hat{\theta }}_{i}$ to refer to estimators of the relationship parameters, and ${\hat{\psi }}_{i}$ to refer to estimators of the corresponding relative parameters.

### Estimation

Equation (1) describes a theoretical population-genetic model, giving an expected relationship between the joint pairwise genotype probabilities, allele probabilities and Jacquard’s coefficients. Equation (1) has been solved with maximum likelihood procedures, by assuming known allele probabilities and building the multinomial likelihood function by multiplying this equation over loci (Laporte et al. 2017). The latter authors estimate the Jacquard coefficients by ML using an EM algorithm, using the crossing design to improve identifiability of the coefficients.

In this article, we elaborate on the alternative approach initiated by Csűrös (2014) and regard Equation (1) as a system of linear equations that could be solved for **Δ** for each pair (*i*, *j*) if **g** and **M** were known; one thus would need to estimate both **g** and **M** from the genotype data, prior to estimating **Δ**. Csűrös (2014) pointed out matrix **M** is structurally singular, and is expected to be of rank seven. It is subject to two linear constraints, both for the columns and the rows. When considering the rows of **M**, it is straightforward to show that these constraints amount to ${{\bf{1}}}^{{\prime} }{\bf{M}}={{\bf{1}}}^{{\prime} }$ and ${{\bf{a}}}^{{\prime} }{\bf{M}}={{\bf{0}}}^{{\prime} }$, with ${{\bf{a}}}^{{\prime} }=(0,-1,-2,1,0,-1,2,1,0)$. Equation (1), viewed as a system of linear equations, can be either consistent or inconsistent, and we address both situations below.

### The consistent system

Equation (1) will constitute a consistent system with infinitely many solutions provided that **M** and **g** are parametrized by exactly the same minor allele probability *p*, and satisfy ${{\bf{a}}}^{{\prime} }{\bf{g}}={{\bf{a}}}^{{\prime} }{\bf{M}}{\mathbf{\Delta }}=0$. In that case, the system can be solved for *some* particular solution either using Gaussian elimination or by the use of a generalized inverse, such as the Moore-Penrose inverse (Searle 1982). Gaussian elimination will reduce **M** to row-echelon form with trailing rows of zeros, and retains the column-sum-one property. Consequently, the obtained Jacquard coefficients will sum to one, but they can be negative. In most cases, **M** will have rank seven due to the two linear restrictions identified by Csűrös (2014). More precisely, the rank of **M** depends on *p* and is at most seven; e.g., **M** will have rank five whenever *p* = *q* = 0.5. Whenever **M** has rank seven, Gaussian elimination leads to ${\hat{\Delta }}_{8}={\hat{\Delta }}_{9}=0$, whereas other coefficients can be negative. This may, at first sight, be surprising, for *Δ*_8_ and *Δ*_9_ typically correspond to the largest Jacquard coefficients found in practice. However, since the order of the variables in the system of equations is arbitrary, Jacquard coefficients can be set to zero at will by permuting the columns of **M** together with their corresponding elements of **Δ**, clearly showing the coefficients are not identified. A consistent linear system with a structurally singular coefficient matrix can also be resolved using the Moore-Penrose inverse (**M**^+^) of **M**, and estimating **Δ** as $\hat{{\mathbf{\Delta }}}={{\bf{M}}}^{+}{\bf{g}}$. This also gives some particular solution with Jacquard coefficients that sum one, but some of them can be negative. The obvious freedom of the coefficients has been parametrized by Csűrös (2014), and his parametrization can be used to map the coefficients to a set of strictly non-negative Jacquard coefficients (i.e., probabilities) with the transformation

26 $$\tilde{{\mathbf{\Delta }}}=\hat{{\mathbf{\Delta }}}+\xi {{\bf{z}}}_{1}+\eta {{\bf{z}}}_{2},$$

where **z**_1_ = (0, 1, 0, −1, 0, −1, −1, 2, 0) and **z**_2_ = (0, 0, 0, 0, 0, 0, 1, 2, 1) + *pq* (−1, −1, 2, 0, 2, 0, −2, 0, 0) and where *ξ* and *η* are real parameters that are constrained by a set of inequalities (Csűrös 2014, Eq. (8)) that warrant the non-negativity of $\tilde{{\mathbf{\Delta }}}$; typically this transformation will also render ${\hat{\Delta }}_{8}$ and ${\hat{\Delta }}_{9}$ non-zero if one attempted to solve the system by Gaussian elimination. Csűrös (2014) used this result to explore the range of variation of the Jacquard coefficients for an empirical pedigree. Here we use this parametrization with molecular marker data to assess the range of variation of the Jacquard coefficients in that setting. An example case is described in Appendix A. However, it should be recognized that observed joint genotype proportions generally do not conform to Equation (1); in particular the condition ${{\bf{a}}}^{{\prime} }{\bf{g}}=0$ is generally not met. For empirical data, Equation (1) will be inconsistent for one cannot merely equate theoretical probabilities with sample statistics. We, therefore, capitalize on the inconsistent case developed below.

### The inconsistent system

For given allele probabilities, the parameters of model (1) can be estimated by averaging, in the unweighted sense, over *L* SNPs such that we need to resolve

27 $$\frac{1}{L}\mathop{\sum }\limits_{l=1}^{L}{{\bf{g}}}_{l}=\frac{1}{L}\mathop{\sum }\limits_{l=1}^{L}{{\bf{M}}}_{l}{\mathbf{\Delta }},$$

which we write concisely as $\overline{{\bf{g}}}=\overline{{\bf{M}}}{\mathbf{\Delta }}$, for **Δ**. In general, this system will be inconsistent, but a best-fitting estimate for **Δ** can be found using a least-squares criterion. Retaining a probabilistic interpretation of the Jacquard coefficients, we minimize the residual sum-of-squares given by

28 $$\sigma ({\mathbf{\Delta }})={\left(\overline{{\bf{g}}}-\overline{{\bf{M}}}{\mathbf{\Delta }}\right)}^{{\prime} }\left(\overline{{\bf{g}}}-\overline{{\bf{M}}}{\mathbf{\Delta }}\right),$$

under the restrictions $\mathop{\sum }\nolimits_{k = 1}^{9}{\Delta }_{k}=1$ and *Δ*_*k*_ ≥ 0. To our best knowledge, this problem has no explicit solution, and we use the R package Rsolnp (Ghalanos and Theussl 2015) to solve it numerically. In our estimation procedure, the averaging of matrix **M**_*l*_ over SNPs implies that higher-order terms of the allele probability like *p*^2^ and *p*^3^ are simply estimated by the average of quadratic and cubic allele probabilities. These estimators are not unbiased (Wang 2022; Weir 1996), but can eventually be corrected for bias due to small sample size. One can thus correct for statistical sampling though this will not correct for genetic sampling (Weir 1996), which makes the expected values of squared allele frequencies, for example, depend on squared allele probabilities plus inbreeding and coancestry values in the sample. No correction for statistical sampling was applied, given the sample size used in our simulations below. We also estimated the Jacquard coefficients of individuals with themselves (${\Delta }_{k}^{(i,i)}$) needed for inbreeding coefficients (see Eqs. (6) and (18)); this estimation was carried out with the additional restriction that only ${\Delta }_{1}^{(i,i)}$ and ${\Delta }_{7}^{(i,i)}$ can be non-zero.

## Simulations

**Fig. 2:**
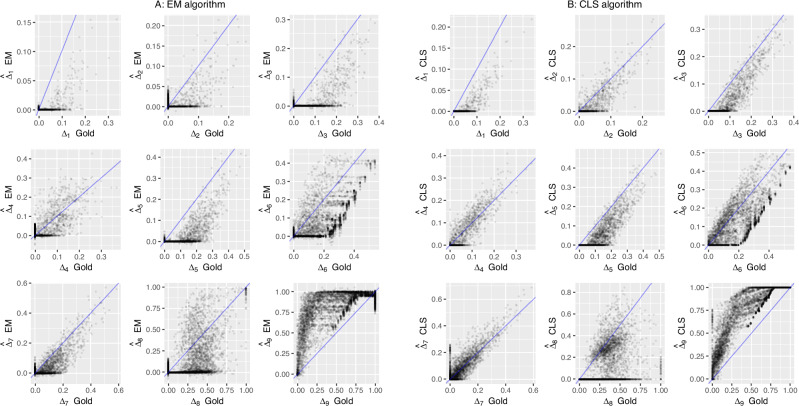
Estimation of gold Jacquard coefficients. **A** EM estimates against the gold values. **B** CLS estimates against the gold values.

We designed a simulation study for assessing the quality of the CLS-based estimator for Jacquard coefficients and derived inbreeding and coancestry coefficients, and compared these with estimates obtained by EM (Laporte et al. 2017). We simulated a pedigree with 20 unrelated founders, 10 males and 10 females, generating seven non-overlapping generations (founders included) totaling 111 individuals using the R-package JGTeach (Goudet 2022). Allele frequencies of the founders were generated by taking independent draws from a Beta(*α* = 1, *β* = 10) distribution, which has positive skew and correspondingly relatively more variants with a low MAF. A picture of the simulated pedigree is shown in Supplementary Fig. S2. For each generation, females had a fertility rate of two, and fifty percent of the males were allowed to breed in order to increase the proportion of related individuals. We generated 20,000 bi-allelic loci on a map of five Morgans (one marker every 0.025 cM). For the simulated pedigree, alleles were dropped along the pedigree by gene dropping using the given recombination map. The number of crossing overs per meiosis was drawn from a Poisson distribution with parameter *λ* equal to the genetic map length, and their positions were drawn from a uniform distribution between 0 and the number of loci minus one (this assumes markers are equally spaced along the genome). These settings allowed a considerable degree of relatedness to build up within a few generations. We found it convenient to use the JGTeach package which is available for the R environment (R Core Team 2023), though other stand-alone softwares are available, notably the SimPedim program by Leal et al. (2005). R instructions for generating the data are given in Appendix C, and the simulated genotype data is also included in R-package Jacquard (Graffelman 2024). In order to assess the quality of the different estimators, we use the root-mean-squared-error (RMSE), which is directly interpretable in the scale of the coefficient of interest. We prefer the RMSE over the use of correlation coefficients, as the latter can be affected by truncation and non-linearity (see Fig. 2). The RMSE can be calculated with respect to the theoretical pedigree values, whose values can be obtained using algorithms like IdCoefs (Abney 2009). However, realized IBD probabilities in a pedigree typically differ from the theoretical values, due to the random nature of meiosis, and given a finite genetic map. We therefore calculated all RMSE statistics with respect to these realized coefficients, which we call gold standard or just gold Jacquard coefficients. Likewise, in RMSE calculations for derived coefficients (inbreeding, coancestry, etc.) we will also use gold values for these coefficients, which are obtained by applying the equations of section “Theory” to the gold standard Jacquard coefficients. By using gold values based on realized IBD rather than expected values of the coefficients according to the pedigree, the extra variation due to deviations from pedigree expectations is avoided. Figure 2 shows scatterplots of the estimated Jacquard coefficients against their gold values for EM and CLS respectively. These plots are very noisy, revealing large errors in particular for estimates ${\hat{\Delta }}_{6},{\hat{\Delta }}_{8}$ and ${\hat{\Delta }}_{9}$, and show it is very hard to estimate the gold Jacquard coefficients reliably. Table 2 reports the RMSE for each coefficient for both methods. This shows the CLS estimates have, over all, a lower RMSE. The table confirms *Δ*_6_, *Δ*_8_ and *Δ*_9_ are most poorly estimated. In relatedness studies it is common practice to filter out low MAF variants. Previous simulation work by Weir and Goudet (2017) has shown this leads to biased estimation of coancestry for the allele-sharing estimator of coancestry. We investigated the effect of MAF filtering by applying three MAF filters for both methods, and considering both the standard Jacquard-coefficient derived ($\hat{\theta }$) and the relative ($\hat{\psi }$) estimates of coancestry and inbreeding. The results in Table 2 show that MAF filtering at one or five percent is detrimental for both the EM and the CLS algorithm, whereas leaving out monomorphic SNPs does not seem to affect the RMSE. The negative effect of MAF filtering is observed for both the standard parameters as well as relative coancestry and inbreeding. The estimates of the relative quantities have, in general, a slightly lower RMSE. Consequently, the plots of our simulation results below used no MAF filter and included all SNPs.

**Table 2:** RMSE of estimated relatedness parameters with respect to the gold Jacquard coefficients and gold relatedness parameters, for EM and CLS.

Method	RMSE Jacquard coefficient									RMSE Relatedness coefficient							
	${\hat{\Delta }}_{1}$	${\hat{\Delta }}_{2}$	${\hat{\Delta }}_{3}$	${\hat{\Delta }}_{4}$	${\hat{\Delta }}_{5}$	${\hat{\Delta }}_{6}$	${\hat{\Delta }}_{7}$	${\hat{\Delta }}_{8}$	${\hat{\Delta }}_{9}$	${\hat{\theta }}_{1}$	${\hat{\theta }}_{2}^{(i)}$	${\hat{\theta }}_{2i}^{(i,j)}$	${\hat{\theta }}_{3}$	${\hat{\theta }}_{4}$	${\hat{\psi }}_{1}$	${\hat{\psi }}_{2}^{(i)}$	${\hat{\psi }}_{2i}^{(i,j)}$
EM All (*n* = 111)	0.022	0.022	0.050	0.033	0.075	0.113	0.046	0.229	0.365	0.110	0.124	0.120	0.243	0.061	0.093	0.114	0.111
EM All *p* founders	0.006	0.016	0.008	0.017	0.012	0.023	0.018	0.056	0.054	0.015	0.017	0.016	0.032	0.008	0.012	0.016	0.015
EM All *p* last gen.	–	–	–	–	–	–	–	–	–	–	–	–	–	–	–	–	–
EM All MAF > 0	0.022	0.022	0.049	0.033	0.075	0.113	0.046	0.229	0.366	0.110	0.124	0.120	0.244	0.061	0.093	0.115	0.111
EM All MAF > 0.01	0.022	0.022	0.050	0.037	0.075	0.115	0.047	0.232	0.373	0.112	0.126	0.123	0.248	0.064	0.095	0.118	0.115
EM All MAF > 0.05	0.023	0.024	0.053	0.063	0.078	0.128	0.052	0.248	0.415	0.123	0.145	0.142	0.277	0.082	0.111	0.146	0.144
CLS All (*n* = 111)	0.018	0.014	0.031	0.018	0.056	0.090	0.039	0.231	0.281	0.077	0.076	0.080	0.164	0.045	0.061	0.067	0.070
CLS All *p* founders	0.011	0.012	0.010	0.013	0.022	0.029	0.031	0.109	0.097	0.023	0.020	0.020	0.045	0.014	0.018	0.019	0.020
CLS All *p* last gen.	0.025	0.027	0.061	0.039	0.093	0.165	0.062	0.308	0.564	0.165	0.173	0.178	0.373	0.082	0.136	0.151	0.155
CLS All reduced	0.018	0.024	0.082	0.165	–	–	0.038	0.221	0.325	0.075	0.072	–	0.187	–	0.059	0.063	–
CLS All MAF > 0	0.018	0.015	0.031	0.018	0.056	0.090	0.040	0.231	0.281	0.077	0.076	0.080	0.164	0.045	0.061	0.066	0.070
CLS All MAF > 0.01	0.019	0.016	0.035	0.019	0.060	0.094	0.040	0.240	0.297	0.082	0.081	0.086	0.176	0.047	0.066	0.071	0.075
CLS All MAF > 0.05	0.028	0.020	0.053	0.069	0.083	0.127	0.040	0.274	0.415	0.124	0.145	0.142	0.267	0.083	0.109	0.153	0.145
CLS All (*n* = 589)	0.001	0.003	0.003	0.007	0.005	0.019	0.014	0.077	0.076	0.015	0.012	0.014	0.035	0.010	0.012	0.012	0.014
CLS All (*n* = 4037)	0.002	0.001	0.002	0.006	0.003	0.008	0.006	0.023	0.021	0.005	0.008	0.008	0.010	0.005	0.005	0.008	0.008

The diagonals of ${\hat{{\mathbf{\Delta }}}}_{1}$ and ${\hat{{\mathbf{\Delta }}}}_{7}$ are not considered in the calculation of the RMSE of the Jacquard coefficients. All: using all individuals in the pedigree; All *p* founders: using all individuals and using founder allele frequencies. All *p* last gen.: using all individuals and allele frequencies of the last generation; All MAF > 0: excluding monomorphic variants; All MAF > 0.01 or > 0.05: using all individuals excluding variants with MAF below the given threshold. All reduced: using all individuals and estimating the reduced system.

**Fig. 3:**
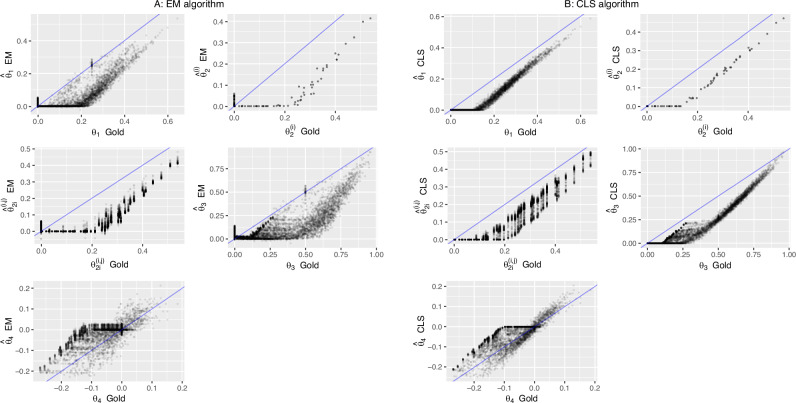
Estimation of relatedness parameters. **A** EM estimates against gold values. **B** CLS estimates against gold values. For inbreeding, both the individual inbreeding coefficients (${\hat{\theta }}_{2}^{(i)}$) and the corresponding pairwise estimates (${\hat{\theta }}_{2i}^{(i,j)}$) are shown.

The poor estimation results are probably in part explained by the fact the coefficients are not identified in the bi-allelic case; we could focus on derived quantities that are identifiable: coancestry, inbreeding and other relatedness parameters (Csűrös 2014). Figure 3 shows scatterplots of the estimated coancestry and inbreeding coefficients against their gold values for EM and CLS respectively, and the corresponding RMSEs are given in the last eight columns of Table 2.

Figure 3 and Table 2 both show that CLS estimates have, in general, less variation and come closer to the *y* = *x* line. However, both estimators substantially underestimate coancestry, inbreeding and the probability that least one IBD pair out of three (*θ*_3_). We repeated the estimation of the Jacquard coefficients and the derived relationship parameters by CLS for two larger pedigrees, the first with 50 male and 50 female founders totaling 589 individuals, and the second with 250 male and 250 female founders totaling 4037 individuals. Plots of all coefficients against their gold values are shown in Figs. S3 and S4. These plots show less noise and diminished bias for the estimation of Jacquard coefficients, coancestry, inbreeding and probability that least one IBD pair out of three, as also reflected by the RMSE calculated for these simulations (see bottom lines of Table 2). However, underestimation of the relationship parameters and coefficients *Δ*_1_, *Δ*_3_, *Δ*_4_, *Δ*_6_ and *Δ*_8_ as well as over-estimation of *Δ*_7_ and *Δ*_9_ is clearly still an issue with a sample of over 500 individuals.

**Fig. 4:**
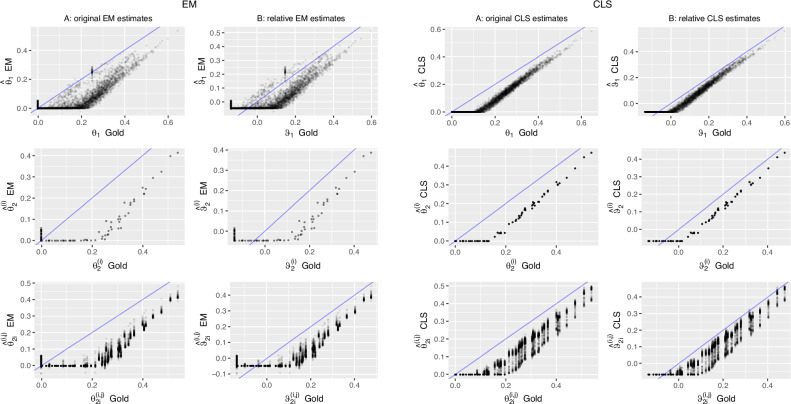
Estimation of gold coancestry and inbreeding coefficients with EM and CLS estimators. The left column (**A**) of each panel shows the original gold values (*θ*_*i*_) against the estimates (${\hat{\theta }}_{i}$) of the algorithm. The right column (**B**) shows the *relative* gold values (*ψ*_*i*_) against their *relative* estimates (${\hat{\psi }}_{i}$). The first row shows coancestry, the second row individual inbreeding coefficients (${\theta }_{2}^{(i)}$) as estimated by ${\rm{diag}}({\hat{{\mathbf{\Delta }}}}_{1})$, the last row shows all pairwise estimates (${\theta }_{2i}^{(i,j)}$) of a given individual obtained according to Eq. (16).

Weir and Goudet (2017, Tables 1 and 3) proposed unbiased allele-sharing estimators for the compound quantities of *relative* coancestry and *relative* inbreeding, which are obtained as follows:

29 $${\hat{\psi }}_{1}=\frac{{A}_{ij}-{A}_{S}}{1-{A}_{S}},\qquad {\hat{\psi }}_{2}=\frac{{A}_{i}-{A}_{S}}{1-{A}_{S}},$$

where *A*_*ij*_ and *A*_*i*_ are allele-sharing statistics, with *A*_*ij*_ the proportion of alleles carried by individuals *i* and *j* that are identical in state (IBS), *A*_*i*_ the proportion of loci for which individual *i* is homozygous, and *A*_*S*_ the average of *A*_*ij*_ over all pairs of distinct individuals (*A*_*S*_ = 1/(*n*(*n* − 1))∑_*i*≠*j*_ *A*_*ij*_). We suggest to convert the EM and CLS estimators for coancestry (${\hat{\theta }}_{1}$) and inbreeding (${\hat{\theta }}_{2}$), which are obtained from the estimated Jacquard coefficients, into estimators of the relative compound quantities, by using a transformation inspired by Eqs. (24) and (29):

30 $${\hat{\psi }}_{1}=\frac{({\hat{\theta }}_{1}-{\hat{\theta }}_{S})}{(1-{\hat{\theta }}_{S})},\qquad {\hat{\psi }}_{2}=\frac{({\hat{\theta }}_{2}-{\hat{\theta }}_{S})}{(1-{\hat{\theta }}_{S})},$$

where ${\hat{\theta }}_{S}$ is the sample average of all pairwise coancestry estimates (${\hat{\theta }}_{S}=\frac{1}{n(n-1)}{\sum }_{i\ne j}{\hat{\theta }}_{ij}$). We note that the allele-sharing estimators directly estimate the relative quantities of interest, whereas Eq. (30) modifies pre-existing estimates of *θ*_1_ and *θ*_2_. The resulting estimators may not be unbiased, though the simulations suggest the relative parameters are better estimated (see the last three columns of Table 2). Moreover, the gold values of *θ*_1_ and *θ*_2_ are clearly underestimated by both algorithms (see Fig. 3); this improves if Eq. (30) is used to estimate the relative gold values (see Fig. 4). We note the sample average of the relative coancestry estimates is zero by construction. We also note the relative estimators amount to a linear rescaling of their original EM and CLS counterparts; consequently any estimator of coancestry will have the same correlation with ${\hat{\theta }}_{1}$ and ${\hat{\psi }}_{1}$; likewise for estimators of inbreeding and ${\hat{\theta }}_{2}$ and ${\hat{\psi }}_{2}$. Figure 4 shows the estimation of the compound relative parameters is more successful for both the EM and the CLS algorithm, and leads to improved estimation of the gold values, as also witnessed by the RMSE statistics in Table 2. We note that gold values for inbreeding are constant across all pairs for a given individual, though the corresponding CLS and EM estimates fluctuate, since not all pairs converge to the same value.

**Fig. 5:**
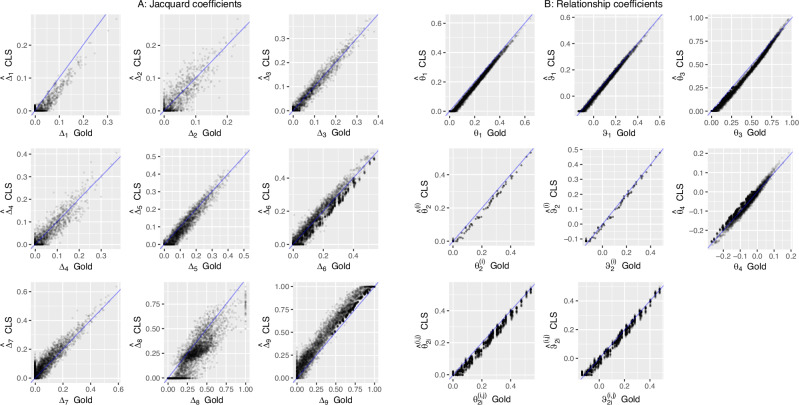
Estimation of gold Jacquard and relationship coefficients by CLS with founder allele frequencies. **A** Estimates of Jacquard coefficients against gold values. **B** Estimates of relationship coefficients against gold values. For inbreeding, both individual estimates (${\hat{\theta }}_{2}^{(i)},{\hat{\psi }}_{2}^{(i)}$, second row) and pairwise estimates (${\hat{\theta }}_{2i}^{(i,j)},{\hat{\psi }}_{2i}^{(i,j)}$, third row) are shown.

The quality of the different estimators depends on the sample allele frequencies and pairs of individuals that are used for comparison. In a simulation, for the estimation of the allele probabilities one can use founders, last-generation individuals or the full pedigree (as in Figs. 2 and 3). When the estimation is carried out using only the last-generation individuals for estimating allele probabilities, RMSE statistics generally deteriorate for CLS, whereas it was mostly impossible to obtain EM estimates in most cases. This is a likely consequence of both a decrease in sample size by 87% and a considerable increase in the percentage of monomorphic SNPs (72% in the last generation, versus 11% in the founder generation), as well as differences between functions of observed allele frequencies and corresponding functions of allele probabilities. When founder allele frequencies are used for estimation, the fit, considering the full pedigree, improves considerably (see Table 2 and Fig. 5), as those frequencies are closer to the relevant allele probabilities. It reduces the RMSE for all Jacquard estimates, and for ${\hat{\Delta }}_{8}$ and ${\hat{\Delta }}_{9}$ in particular, for both the EM and CLS algorithm (see Table 2), and consequently also gives a lower RMSE for the derived relatedness coefficients. With founder allele frequencies, RMSE statistics for the EM algorithm are in general, slightly lower than those of the CLS algorithm, suggesting the allele frequencies are particularly crucial to the EM algorithm. The best estimation results for coancestry and inbreeding are obtained by using the EM algorithm with founder allele frequencies, and estimating the relative quantities that account for average coancestry. We also fitted the reduced bi-allelic system. Supplementary Fig. S5 shows the estimates for the seven reduced Jacquard coefficients and coancestry, inbreeding and ${\hat{\theta }}_{3}$ obtained by fitting the reduced bi-allelic system. Despite fitting two parameters less, the RMSE statistics obtained for coancestry, inbreeding and *θ*_3_ are almost the same as obtained by fitting the nine parameter condensed system.

**Fig. 6:**
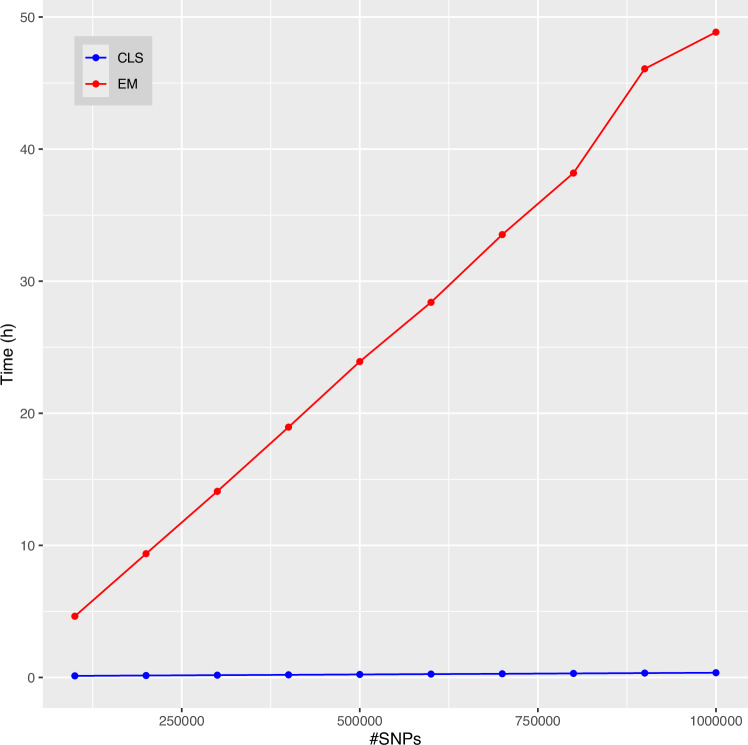
Computational cost (in hours) for estimation of the Jacquard coefficients as a function of the number of bi-allelic SNPs and algorithm used (EM or CLS).

We explored the computational cost of scaling up both algorithms by using increasing numbers of SNPs, up to a million. For the EM algorithm, we found estimation of the full set of Jacquard coefficient to be infeasible for larger numbers of SNPs. Figure 6 shows the CPU time spent for a sample of size 109. For both algorithms the CPU time increases, as expected, linearly with the number of polymorphisms. Figure 6 shows that the CLS algorithm (with tolerance parameter 1E-8) is much faster than the EM algorithm (used with convergence precision 1E-3).

The calculation of the Jacquard coefficients for one million SNPs required 48.86 hours for the EM algorithm, whereas this takes only 0.36 hours for the CLS algorithm, where, for the sake of comparison, we used a single core.

## Discussion

Considerable research effort has been dedicated to the estimation of relationship parameters such as kinship and inbreeding coefficients. There is less work on the estimation of the full set of Jacquard’s genetic identity coefficients with the use of molecular marker data, though interest to do so has clearly increased over the last decade (Guan and Levy 2024; Hanghøj et al. 2019; Korneliussen and Moltke 2015; Zheng et al. 2012). For bi-allelic genetic variants, at first sight there may seem to be little point in reporting the full set because they are not identified (see Fig. S1). Nevertheless, reporting the full set of coefficients is ultimately informative for it will always permit the calculation of any identifiable derived relationship parameter, most interestingly *θ*_3_ and *θ*_4_ given that good estimators for coancestry and inbreeding are available. Maximum likelihood estimation by means of the EM algorithm (Laporte et al. 2017) is computationally very expensive and not feasible on a genomewide scale. The proposed CLS approach is seen to provide comparable estimates of the Jacquard coefficients and derived quantities, and simulations suggest these have smaller RMSE when the founder allele frequencies are unknown. The likelihood approach is probabilistic and multiplies over presumably independent loci, whereas such independence is known not to hold for markers on the same chromosome that are close. The proposed CLS approach averages allele frequencies and joint genotype frequencies over markers but is purely based on least-squares minimization and makes no implicit assumptions about LD.

Our simulations show that the estimation of Jacquard and relationship coefficients works best with founder allele frequencies. Founder allele frequencies will usually be available in breeding programs, but remain unknown in many other empirical settings, where estimation of allele frequencies will typically be based on all available individuals; the latter approach is inevitably affected by the allelic dependencies in the sample. Our simulations are necessarily of limited scope and do not consider the effects of mating system, sex-ratio, genotyping error, depth of the genealogical tree, as well as many other factors. The simulated dataset used in this article focuses on the particularly challenging scenario that combines imprecise allele frequencies (due to the small sample size and the effects of genetic sampling) with strong allelic dependence (high kinship and inbreeding).

The CLS approach proposed in this article is flexible, and can be further extended for variants with multiple alleles, such as microsatellites. In that case, the identification problem of the Jacquard coefficients is resolved if the individuals of a genotype pair are ordered. It is also easily adapted for the classical estimation, under the assumption of no inbreeding, of the Cotterman coefficients. In order to do so, one should just carry out the minimization while restricting the first six Jacquard coefficients to be zero. Additionally, Thompson’s (1976) condition for a genealogically feasible (i.e., pedigree-compatible) relationship may be imposed if desired. A common sense data-analytic strategy is to first estimate the full set of Jacquard coefficients without any inbreeding constraint, and to set the first six to zero in second instance in case no obvious evidence for inbreeding is found. Thompson’s constraint will hold for pedigree-derived coefficients, but not necessarily so for the realized gold values. For empirical data it is not a priori known if the gold values satisfy the constraint. A practical solution is to carry out both minimizations (with and without the constraint) and to choose the best solution of the two. Interestingly, if pairs are known (or believed) to be unrelated, this condition may be imposed by restricting all related states (*Δ*_1_, *Δ*_3_, *Δ*_5_, *Δ*_7_ and *Δ*_8_) to be zero and estimating only *Δ*_9_ and the remaining inbred states. Indeed, any subset of the Jacquard coefficients may be set to zero as suggested by the results of a first exploratory analysis, and to the benefit of reducing the identifiability problem.

Both the EM and CLS algorithms adhere to a strict probabilistic interpretation of Jacquard’s coefficients, their estimates can not be negative and consequently inbreeding and coancestry estimates can neither be negative. The simulations suggest improved approximation of gold inbreeding and coancestry may be possible if negative values would be admitted (see Figs. 3 and 4), though this is clearly less compelling if better estimates of the allele probabilities are available, as is the case with founder allele frequencies (see Fig. 5). Also, the flooring of coancestry estimates at zero pulls estimates of average coancestry towards zero and impacts the correction for average coancestry. EM and CLS algorithms could be further developed towards explicitly estimating the relative quantities of interest, and possibly lifting the non-negativity constraint.

There are some considerations that may be helpful to reduce the computational burden. When the pairs of individuals of interest are known in advance, the expensive calculation of all relationship statistics for all pairs can be avoided. To obtain the statistics of interest, one only needs to calculate the allele frequencies, and subset the calculations of the relationship statistics to the genotype data of the pairs of interest only. This applies to both the CLS and the EM algorithm, as both operate in a pairwise manner. If the estimation of inbreeding is of main interest, for the CLS approach the pairwise calculations can be greatly reduced, because in that case only *n* estimates of the first Jacquard coefficient of an individual are needed instead of the usual $\frac{1}{2}n(n-1)$ pairs. Many genetic studies filter genetic variants by their MAF, with MAF ≤ 0.05 being a commonly used exclusion criterion. Given the typically skewed distribution of the MAF in empirical studies, such filtering implies the exclusion of huge amounts of polymorphisms, and can so reduce computational cost. However, previous simulation work of Weir and Goudet (2017) has shown that MAF filtering introduces bias in the estimation of (pedigree-based) coancestry, whereas our simulations in Table 2 show increased RMSE for all Jacquard coefficients and derived quantities. In the absence of genotyping error, filtering is therefore in principle not appropriate, though it may still be recommended for avoiding sequencing errors, which have been reported to be more frequent among low MAF variants. For both the EM and CLS methods currently written in plain R the computational efficiency can be improved by rewriting the core iterations in C or in Fortran.

Estimation of the Jacquard coefficients with either EM or CLS relies on numerical optimization for which global convergence is not always guaranteed. Proper convergence can be investigated by modifying the tolerance criterion for convergence and by choosing different initial points. If maximum likelihood estimation is preferred, the CLS estimates can be used as initial points for the EM algorithm, to the benefit of the convergence of the latter.

Both the EM algorithm and the proposed CLS approach rely on adequate estimates of the allele probabilities, for which sample allele frequencies are typically used, obtained from either the full data set, or, if possible, from the founder generation only. Reliance on sample allele frequencies is the current approach in relatedness research, as most kinship and inbreeding coefficient estimators do require allele frequency estimates. Alternatively, allele sharing estimators that avoid the use of sample allele frequencies have recently been developed (Goudet et al. 2018; Weir and Goudet 2017). The latter estimators do not provide the full set of Jacquard coefficients, but for estimating coancestry and inbreeding they do not rely on iterative algorithms and are computationally very cheap.

## Software

Estimates of Jacquard’s coefficients with the EM algorithm were obtained with the R package Relatedness (Laporte et al. 2017; Laporte and Mary-Huard 2017). Simulated pedigrees used in this article were generated with R package JGTeach (Goudet 2022). We developed R package Jacquard (Graffelman 2024) which implements estimation of Jacquard’s coefficients and derived quantities by constrained least squares, relying on the optimization functions of R package Rsolnp (Ghalanos and Theussl 2015). In the R environment, Jacquard coefficients can also be estimated by maximum likelihood with the packages SNPRelate (Zheng et al. 2012) and pedsuite (Vigeland 2021). Estimation of Jacquard’s coefficients with next generation sequencing data, while accounting for genotype uncertainty, is possible with the ngsRelate software (Hanghøj et al. 2019; Korneliussen and Moltke 2015). Very recently, a constrained least squares approach has also been proposed by Guan and Levy (2024) and implemented in the C program Kindred.

## Supplementary information

Supplementary information

Software and Dataset 1

## Data Availability

The simulated pedigree data used in the article is available in the R-package Jacquard (Graffelman 2024). Instructions to regenerate the pedigree used in the paper with R-package JGTeach (Goudet 2022) are given in Appendix C.
