# Supplementary material for: Estimation of Jacquard’s genetic identity coefficients with bi-allelic variants by constrained least-squares
Source: Heredity (Edinb). 2024 Nov 7;134(1):10–20. doi: 10.1038/s41437-024-00731-z (PMC11724073; doi:10.1038/s41437-024-00731-z)
Supplement: Supplementary file 1 — Supplementary information [file 41437_2024_731_MOESM1_ESM.pdf]

Supplementary information for

”Estimation of Jacquard’s genetic identity coefficients with bi-allelic variants by constrained least-squares”

Jan Graffelman
Department of Statistics and Operations Research,
Universitat Politècnica de Catalunya
Department of Biostatistics,
University of Washington

Bruce S. Weir
Department of Biostatistics,
University of Washington

Jérôme Goudet
Department of Ecology and Evolution
University of Lausanne

October 21, 2024

Appendix A: Identifiability of Jacquard's coefficients

We illustrate the identifiability problem of the Jacquard coefficients with an example, using the vector of joint genotype probabilities, in the order of Table 1, (0.356, 0.012, 0.038, 0.038, 0.141, 0.033, 0.034, 0.175, 0.173) with associated allele frequency $p = 0.26$. The effective constraints for (ξ, η) are shown as lines in Figure S1A. Any point within the central polygon can map the negative Jacquard coefficients into non-negative probabilities. A feasible point (ξ, η) that produces non-negative Jacquard coefficients can in this case be found by averaging the upper and lower limit of the active constraints, as shown by the single dot in the Figure S1A. By exploring a grid of (ξ, η) values over the central polygon, sets of non-negative Jacquard coefficients are obtained whose range of variation is shown in Figure S1B. This shows a certain variation in these coefficients (Δ_8 in particular), defining sets that are compatible with the same linear system of equations. When the coancestry, inbreeding and other relationship coefficients are calculated from the feasible Jacquard coefficients, Figure S1C is obtained, illustrating that these relationship coefficients are virtually constant and thus identified.

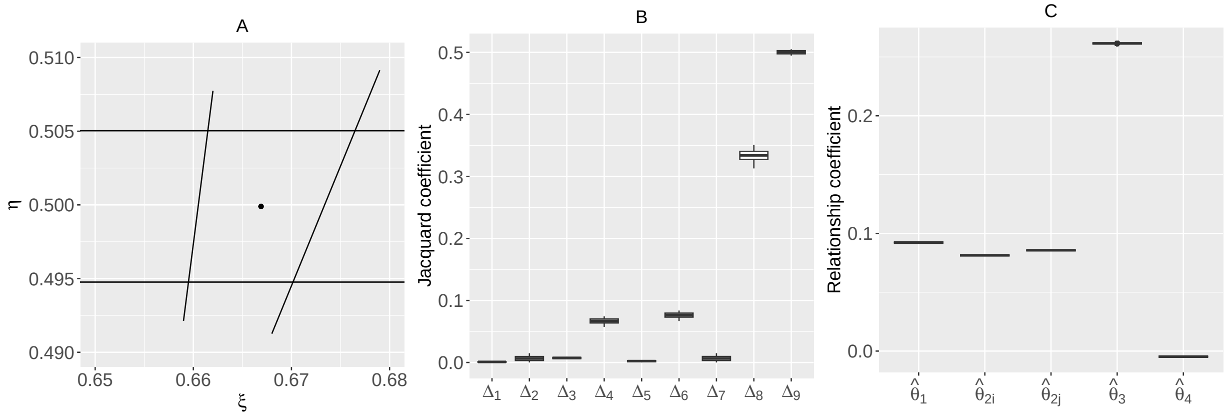

Figure S1: A: Inequalities determining (ξ, η) that map Jacquard coefficients to probabilities. B: Variation in the Jacquard coefficients obtained by varying (ξ, η) over the polygon in panel A. C: Coancestry, inbreeding and other relationship coefficients obtained from the Jacquard coefficients in panel B.

For a bi-allelic system, the Jacquard coefficients could be made identifiable, but this would require the imposition of additional constraints on the coefficients. Alternatively, for a multi-allelic system with pairs of individuals that are ordered, matrix **M** with the conditional joint genotype probabilities, is generally full rank and in that case the Jacquard coefficients are identifiable.

Appendix B: Reduction of the number of Jacquard coefficients

We will use Δ_{35} to represent the sum of Δ_3 and Δ_5; likewise $\Delta_{46} = \Delta_4 + \Delta_6$. We call the new set of seven coefficients the reduced Jacquard coefficients. Importantly, the joining of these columns creates additional linear dependence between the rows of Table 1, where three pairs of rows ((2,4), (3,7) and (6,8)) become identical. We next sum the two rows of each pair, which results in the coefficient matrix of a *biallelic reduced system* given in Table S1. This system retains a linear constraint on the columns, and can again be estimated by constrained least squares with the partly combined Jacquard coefficients having weighted sum one. Again importantly, the joining of the rows precisely eliminates the ordering of the genotype pairs; i.e. the probabilities of (0/0,0/1) and (0/1,0/0) are now summed. As a consequence, the new coefficient matrix $\mathbf{M}^*$ is now of dimension 6×7 and will generally have rank five due to the remaining linear restriction on the sum of the columns; in the exceptional case $p = q = 0.5$ it will have rank four. This fusion leaves the theoretical relatedness parameters θ_1 and θ_3 unaltered. The inbreeding coefficients θ_{2i} and θ_{2j} can no longer be independently estimated, though we can now estimate the probability that either i or j is inbred, and by averaging these probabilities over pairs, an individual inbreeding coefficient can be estimated. Parameter θ_4 (Eq. (8)) can no longer be estimated.

Nr.	Genotype pair	Δ_1	Δ_2	Δ_{35}	Δ_{46}	Δ_7	Δ_8	Δ_9
1	(0/0,0/0)	q	q^2	$2q^2$	$2q^3$	q^2	q^3	q^4
2-4	(0/0,0/1)	0	0	$2pq$	$4pq^2$	0	$2pq^2$	$4pq^3$
3-7	(0/0,1/1)	0	$2pq$	0	$2pq$	0	0	$2p^2q^2$
5	(0/1,0/1)	0	0	0	0	$2pq$	pq	$4p^2q^2$
6-8	(0/1,1/1)	0	0	$2pq$	$4p^2q$	0	$2p^2q$	$4p^3q$
9	(1/1,1/1)	p	p^2	$2p^2$	$2p^3$	p^2	p^3	p^4

Table S1: The biallelic reduced condensed system, consisting of unordered joint genotype probabilities for given IBD patterns and allele probabilities.

For the reduced condensed system, the previously developed matrix equations largely apply with some modifications, in particular

$$\boldsymbol{\theta}_1 = \boldsymbol{\Delta}_1 + \frac{1}{2}\boldsymbol{\Delta}_{35} + \frac{1}{2}\boldsymbol{\Delta}_7 + \frac{1}{4}\boldsymbol{\Delta}_8, \tag{1}$$

and a matrix of inbreeding coefficients is obtained as

$$\boldsymbol{\theta}_2 = \boldsymbol{\Delta}_1 + \boldsymbol{\Delta}_2 + \frac{1}{2}(\boldsymbol{\Delta}_{35} + \boldsymbol{\Delta}_{46}). \tag{2}$$

The previous equations (14), (15) and (19) still apply, and for the newly defined inbreeding coefficients we have that

$$\boldsymbol{\theta}_2 = \boldsymbol{\theta}_2', \quad \text{and} \quad \mathrm{diag}(\boldsymbol{\theta}_2) = \mathrm{diag}(\boldsymbol{\Delta}_1). \tag{3}$$

The probability of at least one pair of IBD alleles among three randomly selected alleles is now given by

$$\boldsymbol{\theta}_3 = \boldsymbol{\Delta}_1 + \boldsymbol{\Delta}_2 + \boldsymbol{\Delta}_{35} + \frac{1}{2}\boldsymbol{\Delta}_{46} + \boldsymbol{\Delta}_7 + \frac{1}{2}\boldsymbol{\Delta}_8. \tag{4}$$

Appendix C: R instructions for the simulation

```
library(JGTeach)
library(hierfstat)

set.seed(1234)
X.ped <- buildped.2sexes(founders.m=10,founders.f=10,fert=2,
                         death.rate=1,breed.prop=0.5,n.tstep=6)

set.seed(123)
PG <- drop.along.ped(X.ped,nloc=20000,maplength=5)

X.gen <- ibd2dos(PG,20)
```

Appendix D: Supplemental figures for the simulations

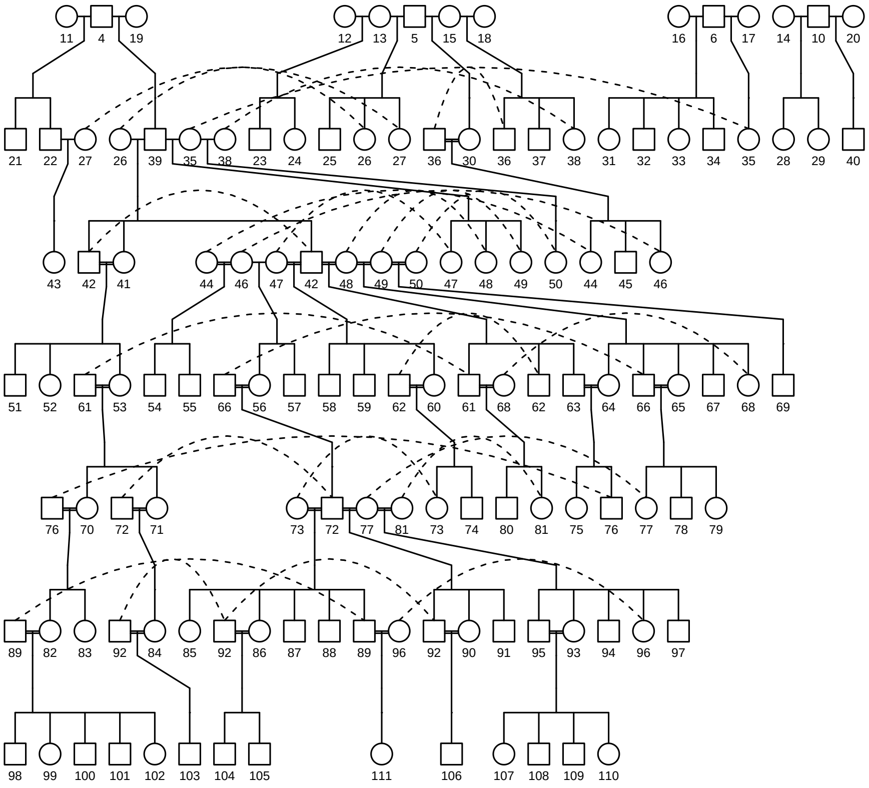

Figure S2: Pedigree simulated by the `JGteach` R-package spanning seven generations (founders included). On average, only 50% of the males breed. Six males of the founder generation are not shown for they left no offspring.

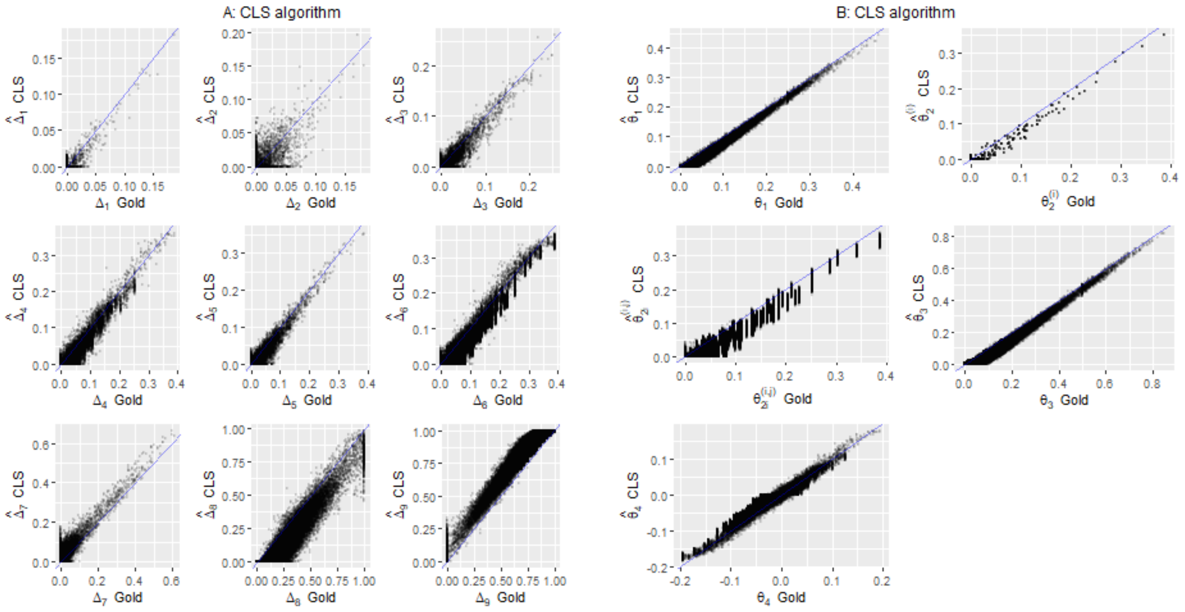

Figure S3: Estimation of Jacquard coefficients and derived relationship coefficients for a sample of 589 individuals using 50 male and 50 female founders. A: Nine condensed Jacquard coefficients versus their gold values. B: Relationship coefficients versus their gold values.

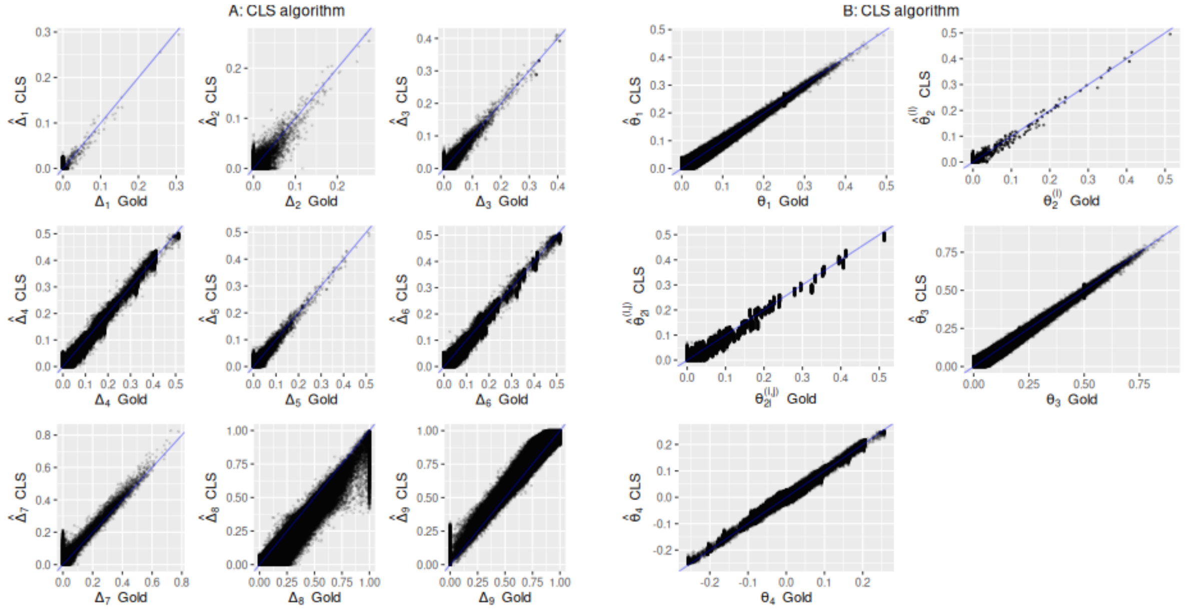

Figure S4: Estimation of Jacquard coefficients and derived relationship coefficients for a sample of 4037 individuals using 250 male and 250 female founders. A: Nine condensed Jacquard coefficients versus their gold values. B: Relationship coefficients versus their gold values.

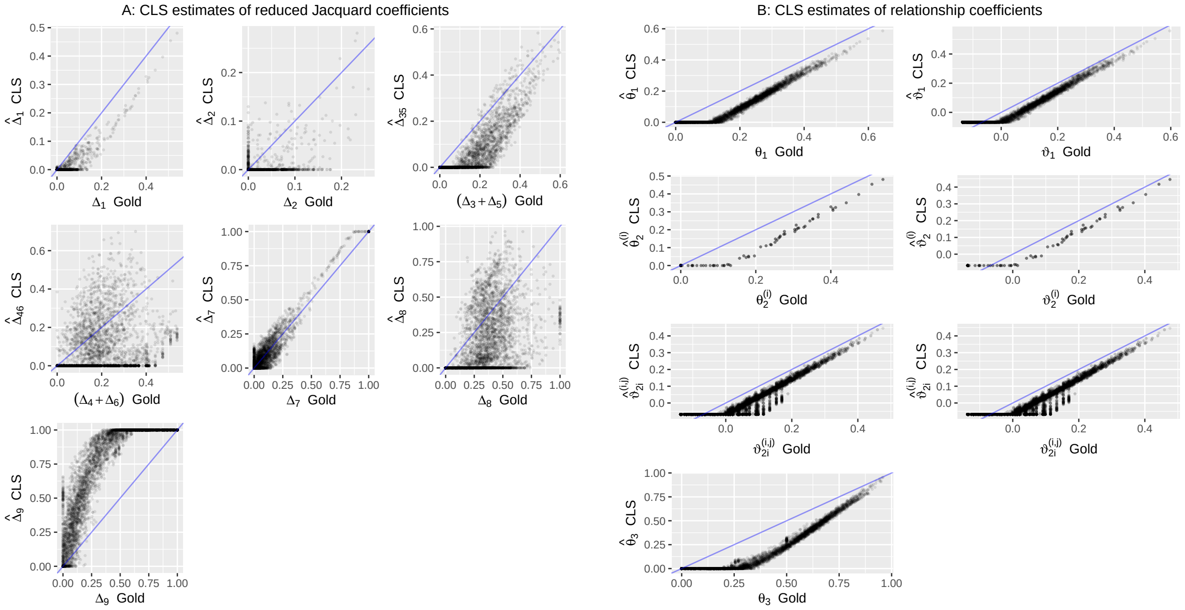

Figure S5: Estimation of reduced Jacquard coefficients. A: Seven reduced Jacquard coefficients versus their gold values. Diagonal elements of $\mathbf{\Delta}_1$ and $\mathbf{\Delta}_7$ are included in the plots of these coefficients. B: Relationship coefficients. The left column represents the original IBD based estimates, the right column the relative estimates.
