# Supplementary material for: Estimation of Jacquard’s genetic identity coefficients with bi-allelic variants by constrained least-squares
Source: Heredity (Edinb). 2024 Nov 7;134(1):10–20. doi: 10.1038/s41437-024-00731-z (PMC11724073; doi:10.1038/s41437-024-00731-z)
Supplement: Supplementary file 2 — Software and Dataset 1 [file 41437_2024_731_MOESM2_ESM.gz › Jacquard/inst/doc/Jacquard.html]

The Jacquard package


# The Jacquard package

#### Jan Graffelman - Dpt. of Statistics, Universitat Politecnica de Catalunya; Dpt. of Biostatistics, University of Washington

#### 2024-09-17

## Introduction

This document explains the basic functionality of the
**Jacquard** package which provides functions for the
estimation of the nine condensed Jacquard coefficients (Jacquard (1974)) for biallelic genetic marker
data using a constrained least squares approach (Graffelman, Weir, and Goudet (2024)).

Outline:

1. Installation
2. Estimation of the Jacquard
   coefficients
3. Estimation of derived relatedness
   parameters
4. Alternative estimation procedures
5. References

## 1. Installation

The package can be installed from CRAN with the instructions

```
#install.packages("Jacquard")
library(Jacquard)
```

## 2. Estimation of Jacquard the coefficients

We illustrate the estimation of the Jacquard coefficients using
(0,1,2) coded genotype data available in the data set
`SimulatedPedigree`, of which we show a small part

```
data(SimulatedPedigree)
SimulatedPedigree[1:5,1:10]
#>       Family ID Father Mother Sex SNP00001 SNP00002 SNP00003 SNP00004 SNP00005
#> ID001      1  1      0      0   1        0        0        0        0        1
#> ID002      1  2      0      0   1        1        2        0        0        0
#> ID003      1  3      0      0   1        0        0        0        0        0
#> ID004      1  4      0      0   1        0        1        0        0        0
#> ID005      1  5      0      0   1        0        1        1        0        0
```

This matrix contains 111 individuals, spanning seven generations, in
its rows and pedigree information plus genotype data of 20,000 SNPs in
its columns. We first separate the pedigree information from the
genotype information.

```
Xped <- SimulatedPedigree[,1:5]
Xgen <- as.matrix(SimulatedPedigree[,6:ncol(SimulatedPedigree)])
```

We next determine the nine joint genotype counts for all pairs of
individuals, storing these counts in a list object with nine lower
triangular matrices, using function `JointGenotypeCounts`.
For efficiency, we here load the precalculated joint counts.

```
#GTC <- JointGenotypeCounts(Xgen)
data(GTC)
names(GTC)
#> [1] "f0000" "f1111" "f1101" "f0111" "f0101" "f1100" "f0011" "f0100" "f0001"
```

E.g., the counts of the major homozygote pairs for the first five
individuals are

```
GTC[[1]][1:5,1:5]
#>       ID001 ID002 ID003 ID004 ID005
#> ID001 16466     0     0     0     0
#> ID002 13987 16507     0     0     0
#> ID003 13982 13965 16440     0     0
#> ID004 13926 13951 13903 16449     0
#> ID005 14000 14063 13977 13979 16556
```

We create a vector with the minor allele frequency of each SNP.

```
mafvec <- mafvector(Xgen)
mafvec[1:5]
#>    SNP00001    SNP00002    SNP00003    SNP00004    SNP00005 
#> 0.004504505 0.063063063 0.045045045 0.000000000 0.013513514
```

We proceed to estimate the Jacquard coefficients by constrained least
squares with function `Jacquard.cls`. For the sake of
illustration, we take the first three founders of the pedigree, and
subset their joint genotype counts

```
ii <- 1:3
Xped[ii,]
#>       Family ID Father Mother Sex
#> ID001      1  1      0      0   1
#> ID002      1  2      0      0   1
#> ID003      1  3      0      0   1

GTCsubset <- list(length = 9)
for (k in 1:9) {
  GTCsubset[[k]] <- matrix(numeric(3^2), ncol = 3)
  GTCsubset[[k]] <- GTC[[k]][ii,ii]
}
```

We set random initial values for the Jacquard coefficients

```
set.seed(123)
delta.init <- runif(9)
delta.init <- delta.init/sum(delta.init)
```

And estimate the pairwise Jacquard coefficients of the three
pairs.

```
output <- Jacquard.cls(GTCsubset,mafvec=mafvec,
                       eps=1e-06,
                       delta.init=delta.init)
#> 3 pairs 20000 SNPs
#> Processing 1 out of 3 
#> Processing 2 out of 3 
#> Processing 3 out of 3
Delta.cls <- output$delta
```

Convergence of the solver can be checked by looking at the field
`convergence`, where 0 indicates proper convergence.

```
output$convergence
#>      [,1] [,2] [,3]
#> [1,]    0    0    0
#> [2,]    0    0    0
#> [3,]    0    0    0
```

Particular estimates of Jacquard coefficients can be extracted from
the `Delta.cls` list object, which is a list of nine
matrices. E.g., \(\Delta\_9\) of the
first pair of individuals (1,2) can be extracted by

```
Delta.cls[[9]][1,2]
#> [1] 0.9999998
```

All nine estimates of the Jacquard coefficients can be obtained with
function `DeltaPair`

```
DeltaPair(Delta.cls,1,2)
#>        J1        J2        J3        J4        J5        J6        J7        J8 
#> 0.0000000 0.0000000 0.0000000 0.0000001 0.0000000 0.0000001 0.0000000 0.0000000 
#>        J9 
#> 0.9999998
```

A pairwise list of the nine Jacquard coefficients of each pair can be
obtained with the function `PairwiseList`.

```
PairwiseList(Delta.cls)
#>             J1 J2 J3 J4 J5 J6 J7 J8 J9
#> ID001-ID002  0  0  0  0  0  0  0  0  1
#> ID001-ID003  0  0  0  0  0  0  0  0  1
#> ID002-ID003  0  0  0  0  0  0  0  0  1
```

The full set of all pairwise Jacquard coefficients can be calculated
with the instructions below. This result is also available in the
precalculated data object `DeltaSimulatedPedigree`.

```
#DeltaSimulatedPedigree <- Jacquard.cls(GTC,mafvec=mafvec,
#                       eps=1e-06,
#                       delta.init=delta.init)$delta
data(DeltaSimulatedPedigree)
```

Boxplots of the estimated Jacquard coefficients can be obtained with
function `BoxplotDelta`. Separate boxplots are shown for the
diagonals of \(\Delta\_1\) and \(\Delta\_7\).

```
BoxplotDelta(DeltaSimulatedPedigree)
```

## 3. Estimation of derived relatedness parameters

The set of five identifiable relatedness parameters dicussed by Csűrös (2014), among them coancestry and
inbreeding coefficients, can be calculated with the function
`CalculateTheta`.

```
Theta <- CalculateTheta(DeltaSimulatedPedigree)
```

E.g., estimates of the kinship coefficients of the first five
(founder) individuals are obtained by

```
Theta[[1]][1:5,1:5]
#>              ID001        ID002        ID003        ID004        ID005
#> ID001 5.000001e-01 3.128504e-08 3.292970e-08 3.989484e-08 3.082953e-08
#> ID002 3.128504e-08 5.000001e-01 2.763879e-08 3.754111e-08 2.416755e-08
#> ID003 3.292970e-08 2.763879e-08 5.000001e-01 4.053117e-08 2.651516e-08
#> ID004 3.989484e-08 3.754111e-08 4.053117e-08 5.000000e-01 2.717388e-08
#> ID005 3.082953e-08 2.416755e-08 2.651516e-08 2.717388e-08 5.000000e-01
```

Individual inbreeding coefficients can be obtained from …

```
diag(Theta[[2]])[1:5]
#>        ID001        ID002        ID003        ID004        ID005 
#> 1.364139e-07 1.239919e-07 1.321129e-07 8.864897e-08 3.719884e-08
diag(DeltaSimulatedPedigree[[1]])[1:5]
#>        ID001        ID002        ID003        ID004        ID005 
#> 1.180065e-07 1.189951e-07 1.110161e-07 8.204957e-08 3.442820e-08
```

Boxplots of identifiable relatedness parameters can be made with
`BoxplotTheta`

```
BoxplotTheta(Theta)
```

## 4. Alternative estimation procedures

There are many estimators for coancestry and inbreeding. Function
`CalculateThetaMom` calculates moment estimators for the five
identifiable relatedness parameters defined by Csűrös (2014).

E.g., moment estimators of the kinship coefficients of the first five
individuals are given by

```
KS.mom[[1]][1:5,1:5]
#>            [,1]       [,2]       [,3]       [,4]       [,5]
#> [1,]  0.4311849 -0.1443478 -0.1436215 -0.1557858 -0.1494313
#> [2,] -0.1443478  0.4337267 -0.1438031 -0.1526993 -0.1312757
#> [3,] -0.1436215 -0.1438031  0.4270091 -0.1645005 -0.1521547
#> [4,] -0.1557858 -0.1526993 -0.1645005  0.4059486 -0.1546964
#> [5,] -0.1494313 -0.1312757 -0.1521547 -0.1546964  0.4279169
```

## 5. References

Csűrös, M. 2014. “Non-Identifiability of Identity Coefficients at
Biallelic Loci.” *Theoretical Population Biology* 92:
22–29. https://doi.org/10.1016/j.tpb.2013.11.001.

Graffelman, J., B. S. Weir, and J. Goudet. 2024. “Estimation of
Jacquard’s Genetic Identity Coefficients with Bi-Allelic
Variants by Constrained Least-Squares.” *Preprint at
bioRxiv*. https://doi.org/10.1101/2024.03.25.586682.

Jacquard, A. 1974. *The Genetic Structure of Populations*.
Springer-Verlag.
